# Supplementary material for: A Likelihood Approach for Real-Time Calibration of Stochastic Compartmental Epidemic Models
Source: PLoS Comput Biol. 2017 Jan 17;13(1):e1005257. doi: 10.1371/journal.pcbi.1005257 (PMC5240920; doi:10.1371/journal.pcbi.1005257)
Supplement: S1 File — (TAR.GZ) [file pcbi.1005257.s014.tar.gz › HSPH_Online-SI-Revision/PF08/n100-mild/n100-mild_PF2_table-8.pdf]

1	newly 3 weeks spec	1329.	784.88	{487., 1173.}	41.9428	0.259647	newly 3 weeks cumul	2670.	1824.88	{1155., 2654.}	34.0172	0.19738	newly 5 weeks spec	2409.	1334.31	{801., 2121.}	45.8551	0.296145	newly 5 weeks cumul	6876.	4194.25	{2693., 6427.}	40.6424	0.249176	newly 60 weeks cumul	33 899.	70 713.8	{60 709., 80 322.}	108.601	0.317437
2	newly 3 weeks spec	1754.	1543.95	{1100., 1946.}	17.6072	0.0882338	newly 3 weeks cumul	4109.	3491.7	{2609., 4338.}	17.8734	0.0903953	newly 5 weeks spec	2989.	2661.91	{1798., 3453.}	19.6096	0.0984665	newly 5 weeks cumul	9407.	8213.16	{5860., 10 447.}	18.22	0.091815	newly 59 weeks cumul	41 142.	75 765.1	{66 054., 82 014.}	84.1552	0.263687
3	newly 3 weeks spec	1224.	1836.41	{864., 3016.}	62.8603	0.201301	newly 3 weeks cumul	2588.	3827.11	{1906., 5919.}	57.3791	0.186375	newly 5 weeks spec	2853.	3640.86	{1504., 6449.}	55.8128	0.208773	newly 5 weeks cumul	7485.	10 132.9	{4541., 16 942.}	55.3687	0.193342	newly 62 weeks cumul	35 853.	76 890.2	{63 556., 91 384.}	114.46	0.326713
4	newly 3 weeks spec	1047.	604.29	{95., 1289.}	64.6791	0.508253	newly 3 weeks cumul	1848.	1129.85	{215., 2301.}	58.678	0.425567	newly 5 weeks spec	3916.	1864.71	{165., 4427.}	74.1596	0.715292	newly 5 weeks cumul	7888.	4089.78	{536., 9593.}	69.3839	0.594841	newly 66 weeks cumul	49 382.	73 075.4	{42 799., 93 227.}	53.012	0.179556
5	newly 3 weeks spec	1604.	895.43	{443., 1810.}	51.5792	0.341517	newly 3 weeks cumul	3273.	1965.1	{1060., 3648.}	46.4565	0.291729	newly 5 weeks spec	2839.	1724.26	{691., 4391.}	57.1906	0.374667	newly 5 weeks cumul	8315.	4956.37	{2286., 10 952.}	52.3422	0.337458	newly 62 weeks cumul	37 686.	67 460.1	{53 026., 89 239.}	79.0057	0.246151
6	newly 3 weeks spec	1697.	1429.3	{957., 2052.}	24.7755	0.127099	newly 3 weeks cumul	3762.	3214.91	{2251., 4389.}	21.8934	0.110435	newly 5 weeks spec	3382.	2501.2	{1507., 3688.}	31.0668	0.176205	newly 5 weeks cumul	9658.	7627.8	{4991., 10 835.}	26.8147	0.144056	newly 60 weeks cumul	39 815.	74 069.4	{63 494., 82 440.}	86.034	0.267329
7	newly 3 weeks spec	621.	578.84	{201., 1190.}	56.351	0.274679	newly 3 weeks cumul	1186.	1153.67	{463., 2255.}	49.5236	0.229485	newly 5 weeks spec	2262.	1465.15	{312., 3573.}	68.0924	0.470309	newly 5 weeks cumul	4678.	3547.63	{1050., 7912.}	61.1127	0.359217	newly 65 weeks cumul	38 409.	75 611.5	{56 375., 91 534.}	96.8588	0.287062
8	newly 3 weeks spec	1914.	958.51	{715., 1152.}	50.1238	0.30971	newly 3 weeks cumul	4004.	2249.85	{1733., 2648.}	44.1801	0.258977	newly 5 weeks spec	3108.	1563.29	{1166., 1915.}	50.3645	0.312949	newly 5 weeks cumul	9610.	5045.78	{3781., 6091.}	47.9621	0.290638	newly 59 weeks cumul	33 244.	70 337.3	{59 670., 77 661.}	111.579	0.32345
9	newly 3 weeks spec	1331.	551.74	{192., 966.}	60.6882	0.477007	newly 3 weeks cumul	2488.	1147.66	{456., 1935.}	55.5957	0.408907	newly 5 weeks spec	3831.	1270.78	{295., 2614.}	69.1611	0.63192	newly 5 weeks cumul	8579.	3264.47	{995., 6083.}	64.1736	0.531255	newly 64 weeks cumul	48 106.	74 090.3	{56 760., 90 419.}	54.0146	0.180916
10	newly 3 weeks spec	2104.	1415.89	{1100., 1733.}	32.7048	0.178292	newly 3 weeks cumul	4740.	3363.71	{2620., 3925.}	29.0357	0.154226	newly 5 weeks spec	3114.	2223.14	{1666., 2772.}	28.7373	0.154588	newly 5 weeks cumul	10 319.	7372.34	{5640., 8798.}	28.5803	0.152287	newly 58 weeks cumul	38 689.	69 636.4	{62 753., 77 914.}	79.9901	0.253698
11	newly 3 weeks spec	890.	487.87	{230., 730.}	45.6011	0.303294	newly 3 weeks cumul	1597.	1100.85	{569., 1593.}	32.9136	0.200747	newly 5 weeks spec	2552.	897.85	{384., 1491.}	64.8178	0.509251	newly 5 weeks cumul	5799.	2662.93	{1276., 4121.}	54.0795	0.380149	newly 62 weeks cumul	44 256.	71 726.	{58 528., 82 268.}	62.0706	0.206986
12	newly 3 weeks spec	1981.	1729.78	{1268., 2133.}	17.6779	0.088641	newly 3 weeks cumul	4456.	4000.51	{3018., 4869.}	15.4576	0.0757423	newly 5 weeks spec	3330.	2791.15	{1885., 3591.}	20.8363	0.108676	newly 5 weeks cumul	10 451.	9025.05	{6618., 11 197.}	18.2079	0.0923357	newly 58 weeks cumul	44 615.	72 445.5	{66 093., 80 597.}	62.3793	0.209359
13	newly 3 weeks spec	1116.	503.03	{222., 780.}	58.8056	0.42266	newly 3 weeks cumul	2037.	1087.21	{523., 1574.}	51.268	0.337525	newly 5 weeks spec	3104.	1033.91	{388., 1717.}	69.8495	0.589427	newly 5 weeks cumul	7098.	2850.49	{1204., 4541.}	63.4608	0.482591	newly 63 weeks cumul	40 666.	74 487.3	{64 051., 83 830.}	83.1686	0.260085
14	newly 3 weeks spec	1519.	602.49	{194., 1139.}	65.47	0.532155	newly 3 weeks cumul	2463.	1211.36	{469., 2200.}	57.7393	0.419649	newly 5 weeks spec	4569.	1442.18	{317., 3180.}	72.8024	0.695268	newly 5 weeks cumul	9906.	3602.29	{1031., 7256.}	68.5682	0.590391	newly 65 weeks cumul	48 448.	72 285.6	{55 370., 90 851.}	49.306	0.166622
15	newly 3 weeks spec	1182.	514.69	{263., 804.}	56.7098	0.403912	newly 3 weeks cumul	2013.	1157.2	{613., 1708.}	43.6612	0.280588	newly 5 weeks spec	3065.	959.56	{436., 1584.}	68.693	0.561151	newly 5 weeks cumul	7143.	2824.57	{1355., 4366.}	60.6234	0.44931	newly 62 weeks cumul	48 233.	73 720.3	{64 017., 83 083.}	52.8421	0.182195
16	newly 3 weeks spec	1433.	1164.51	{920., 1545.}	22.7111	0.113442	newly 3 weeks cumul	3454.	2748.19	{2213., 3694.}	23.4016	0.117897	newly 5 weeks spec	2363.	1841.39	{1406., 2519.}	25.7863	0.132245	newly 5 weeks cumul	7772.	6059.82	{4716., 8288.}	25.1369	0.128225	newly 58 weeks cumul	32 590.	70 429.7	{62 744., 76 447.}	116.108	0.33343
17	newly 3 weeks spec	933.	593.12	{264., 1007.}	45.6184	0.298328	newly 3 weeks cumul	1663.	1247.66	{621., 1973.}	38.7974	0.226144	newly 5 weeks spec	2774.	1317.25	{411., 2666.}	57.8381	0.462794	newly 5 weeks cumul	6118.	3458.37	{1362., 6201.}	50.4153	0.356554	newly 64 weeks cumul	46 517.	72 496.5	{53 660., 90 698.}	55.8495	0.184856
18	newly 3 weeks spec	223.	733.22	{22., 1664.}	300.386	0.681519	newly 3 weeks cumul	346.	1169.13	{35., 2439.}	289.731	0.622313	newly 5 weeks spec	1979.	2509.33	{24., 8957.}	151.345	1.0326	newly 5 weeks cumul	3142.	5216.26	{95., 15 185.}	174.156	0.868754	newly 68 weeks cumul	44 313.	62 763.6	{20 057., 90 248.}	69.6045	0.487445
19	newly 3 weeks spec	1287.	903.05	{679., 1129.}	31.6216	0.170786	newly 3 weeks cumul	2744.	2127.02	{1620., 2662.}	25.4687	0.130962	newly 5 weeks spec	2837.	1463.86	{1077., 1837.}	48.4773	0.299004	newly 5 weeks cumul	7629.	4749.82	{3622., 5800.}	38.3002	0.217699	newly 59 weeks cumul	38 174.	71 793.	{62 025., 76 902.}	88.0678	0.273145
20	newly 3 weeks spec	216.	537.29	{22., 1184.}	217.19	0.62634	newly 3 weeks cumul	340.	892.11	{35., 1949.}	211.191	0.568923	newly 5 weeks spec	1268.	1886.34	{24., 5827.}	165.475	0.910741	newly 5 weeks cumul	2188.	3894.53	{95., 10 550.}	176.217	0.757722	newly 68 weeks cumul	40 762.	61 137.9	{22 643., 89 885.}	76.2647	0.484766
21	newly 3 weeks spec	570.	610.29	{22., 1256.}	121.24	0.789333	newly 3 weeks cumul	880.	989.73	{35., 2026.}	110.79	0.672097	newly 5 weeks spec	2390.	2100.06	{24., 6444.}	123.329	1.05325	newly 5 weeks cumul	4463.	4362.74	{95., 12 324.}	122.345	0.90446	newly 68 weeks cumul	44 593.	63 547.6	{24 289., 90 826.}	67.9308	0.428255
22	newly 3 weeks spec	1853.	1992.36	{1587., 2467.}	15.02	0.0609796	newly 3 weeks cumul	4544.	4620.38	{3784., 5450.}	12.3605	0.0529336	newly 5 weeks spec	2912.	3203.16	{2392., 3944.}	18.114	0.0724174	newly 5 weeks cumul	9679.	10 400.8	{8232., 12 662.}	15.4029	0.0626865	newly 58 weeks cumul	43 141.	73 096.2	{64 707., 80 976.}	69.4356	0.22776
23	newly 3 weeks spec	936.	490.76	{208., 916.}	54.812	0.375585	newly 3 weeks cumul	1680.	1080.29	{495., 1953.}	46.1494	0.282435	newly 5 weeks spec	2751.	960.82	{296., 1868.}	68.956	0.582102	newly 5 weeks cumul	5966.	2726.46	{1035., 5185.}	60.361	0.442155	newly 63 weeks cumul	45 926.	67 885.9	{51 984., 80 091.}	47.8157	0.163619
24	newly 3 weeks spec	678.	745.2	{71., 1409.}	112.024	0.543949	newly 3 weeks cumul	1172.	1259.09	{167., 2417.}	97.4855	0.455955	newly 5 weeks spec	2554.	2070.93	{104., 4932.}	103.857	0.746719	newly 5 weeks cumul	4980.	4713.53	{387., 10 346.}	106.525	0.621418	newly 67 weeks cumul	42 361.	72 803.9	{49 102., 92 502.}	72.5828	0.225753
25	newly 3 weeks spec	1220.	1123.49	{896., 1349.}	13.4746	0.0638743	newly 3 weeks cumul	2798.	2635.08	{2143., 3108.}	11.9149	0.0552478	newly 5 weeks spec	2397.	1825.91	{1451., 2224.}	23.9328	0.124875	newly 5 weeks cumul	7013.	5912.75	{4822., 7030.}	16.7804	0.0837156	newly 58 weeks cumul	41 718.	74 392.9	{69 887., 76 569.}	78.3234	0.250881
26	newly 3 weeks spec	1422.	908.24	{583., 1373.}	37.9466	0.224371	newly 3 weeks cumul	2792.	2082.44	{1372., 2984.}	30.0501	0.164423	newly 5 weeks spec	3228.	1555.02	{945., 2582.}	52.0483	0.347294	newly 5 weeks cumul	8236.	4836.79	{3065., 7426.}	42.0059	0.257186	newly 60 weeks cumul	43 633.	72 563.	{63 600., 78 958.}	66.3031	0.219466
27	newly 3 weeks spec	1429.	835.26	{684., 961.}	41.5493	0.237554	newly 3 weeks cumul	3015.	1971.74	{1631., 2299.}	34.6023	0.188086	newly 5 weeks spec	2878.	1368.13	{1102., 1609.}	52.4625	0.327712	newly 5 weeks cumul	7889.	4417.63	{3677., 5098.}	44.0027	0.255749	newly 58 weeks cumul	41 619.	73 410.1	{71 780., 75 407.}	76.386	0.24632
28	newly 3 weeks spec	1395.	1262.81	{802., 1656.}	20.3534	0.101201	newly 3 weeks cumul	3462.	2920.26	{2032., 3710.}	19.6857	0.102203	newly 5 weeks spec	2612.	2099.51	{1286., 3056.}	26.4897	0.143905	newly 5 weeks cumul	8023.	6666.68	{4399., 9120.}	22.864	0.120452	newly 59 weeks cumul	37 337.	71 230.4	{62 515., 81 037.}	90.777	0.278284
29	newly 3 weeks spec	1153.	972.52	{603., 1469.}	29.4935	0.152325	newly 3 weeks cumul	2468.	2164.65	{1493., 3086.}	25.5028	0.126864	newly 5 weeks spec	2181.	1786.53	{994., 2820.}	34.2004	0.182341	newly 5 weeks cumul	6228.	5283.37	{3348., 8040.}	30.1636	0.154806	newly 61 weeks cumul	30 741.	72 995.1	{62 896., 82 612.}	137.452	0.373039
30	newly 3 weeks spec	1065.	339.4	{140., 523.}	68.5709	0.554242	newly 3 weeks cumul	1901.	772.73	{343., 1121.}	60.0037	0.438958	newly 5 weeks spec	3590.	618.8	{236., 1091.}	82.7632	0.847656	newly 5 weeks cumul	7402.	1851.07	{767., 2965.}	75.0942	0.665016	newly 63 weeks cumul	47 995.	67 712.9	{53 377., 79 626.}	41.198	0.14565
31	newly 3 weeks spec	1303.	674.63	{509., 845.}	48.2249	0.297764	newly 3 weeks cumul	2596.	1570.5	{1115., 1916.}	39.6518	0.229629	newly 5 weeks spec	2456.	1124.03	{798., 1412.}	54.2333	0.353329	newly 5 weeks cumul	6882.	3570.59	{2596., 4428.}	48.117	0.296795	newly 60 weeks cumul	37 472.	71 423.7	{64 265., 77 334.}	90.6055	0.278783
32	newly 3 weeks spec	2005.	1855.7	{1450., 2235.}	13.5461	0.0651662	newly 3 weeks cumul	4624.	4317.88	{3400., 5000.}	11.83	0.0562957	newly 5 weeks spec	3092.	2995.32	{2136., 3736.}	15.8545	0.0736022	newly 5 weeks cumul	10 135.	9708.23	{7384., 11 709.}	13.653	0.063841	newly 58 weeks cumul	42 820.	72 927.5	{65 291., 81 165.}	70.3118	0.22992
33	newly 3 weeks spec	116.	684.84	{22., 1540.}	531.776	0.638773	newly 3 weeks cumul	183.	1109.11	{35., 2439.}	536.191	0.664456	newly 5 weeks spec	1068.	2291.96	{24., 8835.}	225.363	0.882342	newly 5 weeks cumul	1629.	4816.27	{95., 15 185.}	279.772	0.72043	newly 68 weeks cumul	33 313.	63 108.3	{24 289., 90 826.}	108.026	0.494201
34	newly 3 weeks spec	1093.	525.86	{154., 1006.}	56.0714	0.443394	newly 3 weeks cumul	1962.	1080.31	{353., 1985.}	50.1024	0.363399	newly 5 weeks spec	3687.	1280.72	{256., 2534.}	67.4402	0.642452	newly 5 weeks cumul	7846.	3194.61	{817., 6141.}	61.786	0.529719	newly 64 weeks cumul	44 642.	76 402.7	{58 483., 90 933.}	71.1453	0.227162
35	newly 3 weeks spec	20.	584.22	{14., 1540.}	2836.3	1.16251	newly 3 weeks cumul	142.	980.35	{32., 2294.}	616.134	0.778423	newly 5 weeks spec	625.	2314.56	{24., 8835.}	350.278	0.809537	newly 5 weeks cumul	970.	4564.04	{95., 15 083.}	423.682	0.727453	newly 68 weeks cumul	40 997.	63 963.9	{22 643., 90 826.}	79.1711	0.497599
36	newly 3 weeks spec	277.	530.35	{22., 1184.}	173.664	0.6494	newly 3 weeks cumul	444.	880.72	{35., 1949.}	159.658	0.565526	newly 5 weeks spec	1470.	1869.79	{24., 5827.}	149.976	0.941178	newly 5 weeks cumul	2688.	3855.91	{95., 10 550.}	150.915	0.791005	newly 68 weeks cumul	33 226.	60 957.9	{15 133., 90 082.}	105.89	0.534768
37	newly 3 weeks spec	1497.	786.97	{419., 1110.}	47.5197	0.308318	newly 3 weeks cumul	2865.	1768.86	{1010., 2391.}	38.9089	0.235727	newly 5 weeks spec	3432.	1407.98	{671., 2202.}	58.9749	0.427993	newly 5 weeks cumul	8555.	4242.32	{2274., 6176.}	50.4554	0.336359	newly 61 weeks cumul	44 446.	72 607.8	{62 685., 82 174.}	63.3618	0.211036
38	newly 3 weeks spec	250.	639.65	{14., 1540.}	234.268	0.678656	newly 3 weeks cumul	547.	1031.26	{32., 2294.}	162.724	0.6291	newly 5 weeks spec	1505.	2287.1	{24., 8835.}	172.477	0.978923	newly 5 weeks cumul	2547.	4676.51	{95., 15 083.}	187.755	0.829199	newly 68 weeks cumul	38 637.	61 545.	{15 133., 90 248.}	85.5766	0.515503
39	newly 3 weeks spec	1338.	669.59	{205., 1291.}	59.7825	0.452648	newly 3 weeks cumul	2487.	1347.31	{493., 2530.}	53.6506	0.380269	newly 5 weeks spec	3912.	1648.68	{320., 3712.}	69.8292	0.625663	newly 5 weeks cumul	8892.	4073.4	{1059., 8370.}	64.4017	0.521189	newly 65 weeks cumul	39 846.	66 721.1	{42 210., 91 984.}	68.3544	0.210232
40	newly 3 weeks spec	586.	768.25	{86., 1664.}	117.903	0.459667	newly 3 weeks cumul	886.	1318.69	{195., 2749.}	113.888	0.367098	newly 5 weeks spec	3300.	2100.28	{139., 6243.}	91.6267	0.759219	newly 5 weeks cumul	5752.	4812.23	{430., 11 911.}	96.8016	0.607755	newly 67 weeks cumul	45 872.	72 801.4	{46 323., 92 932.}	60.6701	0.196572
41	newly 3 weeks spec	397.	603.42	{22., 1249.}	152.096	0.713648	newly 3 weeks cumul	582.	982.62	{35., 1949.}	144.636	0.594059	newly 5 weeks spec	2106.	2053.66	{24., 6228.}	129.953	1.01882	newly 5 weeks cumul	3564.	4287.31	{95., 11 248.}	136.662	0.845177	newly 68 weeks cumul	48 915.	62 827.8	{23 478., 90 082.}	57.3225	0.40954
42	newly 3 weeks spec	795.	420.4	{133., 678.}	59.3031	0.421779	newly 3 weeks cumul	1426.	863.54	{352., 1428.}	51.777	0.339352	newly 5 weeks spec	2995.	1015.96	{241., 1739.}	74.3793	0.677573	newly 5 weeks cumul	5937.	2542.57	{802., 3951.}	67.0738	0.531107	newly 65 weeks cumul	44 109.	72 182.2	{55 087., 90 865.}	63.645	0.206411
43	newly 3 weeks spec	339.	748.31	{22., 1540.}	211.26	0.698944	newly 3 weeks cumul	542.	1197.39	{44., 2439.}	192.585	0.603904	newly 5 weeks spec	1978.	2376.67	{44., 8835.}	145.195	0.989614	newly 5 weeks cumul	3344.	5087.49	{125., 15 083.}	161.887	0.832147	newly 68 weeks cumul	47 323.	63 813.3	{24 289., 90 248.}	61.2859	0.416214
44	newly 3 weeks spec	386.	946.45	{90., 1363.}	217.837	0.419771	newly 3 weeks cumul	600.	1506.	{221., 2266.}	197.76	0.330958	newly 5 weeks spec	2455.	2279.82	{144., 5026.}	119.695	0.740791	newly 5 weeks cumul	4090.	5500.71	{462., 8797.}	142.296	0.572578	newly 67 weeks cumul	47 913.	69 447.2	{42 265., 92 715.}	49.3901	0.165913
45	newly 3 weeks spec	1342.	1059.49	{410., 1738.}	41.3689	0.244967	newly 3 weeks cumul	2662.	2195.11	{962., 3487.}	36.2415	0.203824	newly 5 weeks spec	2863.	2286.22	{665., 4196.}	50.5945	0.30972	newly 5 weeks cumul	7410.	6067.78	{2154., 10 468.}	44.5074	0.257955	newly 63 weeks cumul	42 303.	74 369.4	{57 224., 90 615.}	75.8018	0.237202
46	newly 3 weeks spec	594.	473.8	{122., 965.}	53.8889	0.308936	newly 3 weeks cumul	1181.	983.01	{284., 1918.}	47.1389	0.259145	newly 5 weeks spec	2667.	1091.85	{189., 2538.}	64.0731	0.576055	newly 5 weeks cumul	5207.	2798.9	{615., 6115.}	57.0716	0.431988	newly 64 weeks cumul	48 956.	74 508.5	{58 175., 90 578.}	52.1947	0.176488
47	newly 3 weeks spec	763.	446.72	{175., 863.}	51.4522	0.359425	newly 3 weeks cumul	1478.	932.53	{399., 1673.}	46.5217	0.304952	newly 5 weeks spec	3193.	997.83	{271., 2298.}	71.0783	0.66916	newly 5 weeks cumul	6436.	2601.12	{880., 5444.}	62.5295	0.51478	newly 64 weeks cumul	48 321.	72 361.	{58 064., 89 975.}	49.7507	0.168901
48	newly 3 weeks spec	1223.	650.03	{522., 766.}	46.8496	0.279816	newly 3 weeks cumul	2426.	1531.39	{1239., 1756.}	36.8759	0.204371	newly 5 weeks spec	3101.	1094.21	{856., 1316.}	64.7143	0.458414	newly 5 weeks cumul	7512.	3474.56	{2749., 4100.}	53.7465	0.339967	newly 59 weeks cumul	44 170.	73 470.9	{70 275., 75 744.}	66.3367	0.22078
49	newly 3 weeks spec	820.	561.85	{141., 1211.}	64.6476	0.408951	newly 3 weeks cumul	1602.	1130.78	{345., 2258.}	56.7541	0.342667	newly 5 weeks spec	2523.	1385.38	{214., 3578.}	74.8751	0.606814	newly 5 weeks cumul	5593.	3417.84	{739., 8014.}	68.6165	0.482235	newly 65 weeks cumul	35 659.	66 445.6	{42 151., 91 931.}	86.5379	0.253223
50	newly 3 weeks spec	648.	523.08	{119., 1220.}	65.5463	0.38854	newly 3 weeks cumul	1146.	1007.9	{287., 2197.}	58.8813	0.318387	newly 5 weeks spec	2869.	1458.08	{190., 3295.}	76.4601	0.662144	newly 5 weeks cumul	5616.	3360.25	{662., 7675.}	70.4824	0.521513	newly 66 weeks cumul	40 068.	70 162.3	{46 438., 92 545.}	75.7032	0.23128
